# Disturbed rest-activity rhythm in Parkinson's disease: Associations with motor severity and orthostatic hypotension

**DOI:** 10.1177/1877718X251388890

**Published:** 2025-10-21

**Authors:** Manon D Mijnsbergen, Vasileios Exadaktylos, Jacobus J van Hilten, Dagmar H Hepp, Roel HA Weijer

**Affiliations:** 1 Department of Neurology, Leiden University Medical Center, Leiden, the Netherlands; 2 Centre for Human Drug Research, Leiden, the Netherlands; 3 Department of Rehabilitation Medicine, Amsterdam University Medical Center, VUmc, Amsterdam Movement Sciences, Amsterdam, the Netherlands

**Keywords:** Parkinson's disease, wearable device, diurnal rhythm, remote patient monitoring

## Abstract

**Background:**

The rest-activity rhythm (RAR) captures the distribution of rest and activity periods between and within days. Disturbed RAR has been observed in Parkinson's disease (PD), but the contribution of motor and non-motor symptoms to RAR disturbances remains unclear.

**Objective:**

To evaluate the extent to which motor and non-motor symptoms account for variations in RAR between people with PD (PwPD).

**Methods:**

464 PwPD and 105 age-matched controls of the ProPark cohort underwent assessment of motor, psychiatric, sleep, and autonomic function. Participants wore a wrist motion sensor for one week to measure RAR, i.e., relative amplitude, interdaily stability, and intradaily variability. Associations between RAR, and demographic and clinical variables were examined using backward stepwise regression models.

**Results:**

PwPD had lower relative amplitude (p < 0.001), lower interdaily stability (p < 0.001), and higher intradaily variability (p < 0.001), than healthy controls. Motor impairment (β=-0.262, 95% CI = [-0.487,-0.125], R²=6.8%) and the presence of orthostatic hypotension (OH) (β=-0.142, 95% CI = [-0.276,-0.026], R²=1.9%) were associated with lower relative amplitude. Motor impairment (β=0.129, 95% CI = [0.005,0.238], R²=2.5%), the presence of OH (β=0.182, 95% CI = [0.079,0.307], R²=3.6%), and higher age (β=0.158, 95% CI = [0.039,0.277], R²=4.0%) were associated with higher intradaily variability, while female gender (β=-0.196, 95% CI = [-0.318,-0.088], R²=4.7%) was associated with lower intradaily variability. Female gender was linked to higher interdaily stability (β=0.205, 95% CI = [0.071,0.321], R²=4.2%).

**Conclusions:**

More severe motor impairment and having OH are associated with RAR disturbances in PwPD. Future studies are needed to evaluate whether optimizing treatment of motor impairment and OH, both symptomatic and asymptomatic, can improve RAR and increase mobility for PwPD.

## Plain language summary

In this study, we used motion sensors to track the daily activity patterns of people with Parkinson's disease (PD). These patterns, called the rest-activity rhythm, show how periods of rest and physical activity are distributed throughout a 24-h day. Disruptions in this rhythm, such as having more rest breaks during the day, can lead to negative health consequences such as accelerated biological aging. While people with PD may also experience these disruptions, the relationship to PD symptoms is not well understood. To explore this, we are studying 464 people with PD, and 105 healthy controls from the Profiling Parkinson (ProPark) study. Participants completed questionnaires, had a clinical examination, and wore motion sensors at home for a week. The results showed that, compared to healthy controls, people with PD were generally less active during the day, took more breaks, and had a more varied daily rhythm. We also found that worse motor symptoms and orthostatic hypotension (a drop in blood pressure that happens when standing up), were linked to a more disrupted rest-activity rhythm. Interestingly, 85% of the participants with PD and orthostatic hypotension did not have related symptoms such as dizziness when standing up, but it still affects their activity patterns. This highlights the need to routinely screen for orthostatic hypotension in clinical practice, even when patients are asymptomatic. Addressing both motor symptoms and orthostatic hypotension may help people with PD to maintain their daily activity patterns.

## Introduction

Physical activity is known to protect against chronic health problems such as cardiovascular disease, and diabetes.^1^ In Parkinson's disease (PD), physical activity can also improve clinical outcomes, including better gait stability and performance of activities of daily living (ADL).^2^ Therefore, the commonly observed decline in physical activity levels in people with Parkinson's disease (PwPD) may be an important target for interventions.^3^

Physical activity in PwPD is traditionally evaluated using self-reported data. However, this approach can be inaccurate due to over- or underestimation of physical activity.^4^ An alternative approach to self-reporting is the use of wearable sensors, which provide an objective method to monitor daily functioning by continuously measuring physical activity.^5^ Quantitative parameters of physical activity, derived from these sensors, include measures such as daily step count or time spent in low and moderate to vigorous activity.^6^ Since motor function in PwPD may show daily fluctuations, clinically relevant information may be present in the distribution and duration of periods of activity and rest within and between days, better known as the rest-activity rhythm (RAR).^5–7^

In healthy adults, having disturbances in RAR is associated with accelerated biological aging,^8^ and all-cause mortality.^9^ RAR disturbances have also previously been shown in PwPD,^5,10–13^ indicating that there are disease-related limitations that make it difficult for PwPD to maintain mobility and the performance of ADL, potentially impacting quality of life.^14^ Previous studies on the associations between PD symptoms and RAR are characterized by small sample sizes and a narrow focus on a single clinical symptom, such as hallucinations,^10^ heart rate variability,^11^ and cognitive impairment.^12^ A better understanding of how the broad spectrum of motor and non-motor symptoms in PD influence RAR dysfunction is essential for the development of new personalized treatment approaches. To address this issue, we evaluated the RAR in a large cohort of PwPD and healthy controls. We aimed to validate that RAR is disturbed in PD compared to healthy controls in this cohort, and to examine which, and to what extent, motor and non-motor symptoms are associated with RAR disturbances.

## Methods

This study is part of the Profiling Parkinson's disease (ProPark) study, a longitudinal multicenter cohort study consisting of individuals clinically diagnosed with PD and a healthy control group. This study was approved by the medical ethical committee of the Amsterdam University Medical Center, reference number 2019-515. All participants provided informed consent prior to enrollment.

### Participants

For ProPark, PwPD were recruited at three university medical centers (i.e., Leiden University Medical Center, Amsterdam University Medical Center and Erasmus Medical Center) and a community-based hospital (i.e., Meander Medical Center). In addition, PwPD and healthy controls were recruited via advertisements in magazines and newsletters (e.g., from the Dutch Parkinson Patient Association), and flyers and posters available at all university medical centers and the community-based hospital mentioned above. The inclusion criteria for PwPD were: a PD diagnosis by a neurologist according to the Movement Disorder Society clinical diagnostic criteria for PD,^15^ a disease duration of ≤15 years, aged ≥18 years, and able to read and understand Dutch. Exclusion criteria were: a score of ≤16 on the Montreal Cognitive Assessment (MoCA) scale,^16^ currently on advanced therapy (i.e., levodopa continuous intestinal gel, apomorphine treatment or Deep Brain Stimulation), presence of co-morbidities that would hamper interpretation of parkinsonian disability in the opinion of the investigator, and unwillingness to be informed of unexpected medical findings. PwPD who are receiving advanced therapies are excluded because the primarily aim of the ProPark study is to examine disease progression and adverse drug reactions in relation to biomarkers. Advanced therapies can significantly impact adverse drug reactions and interfere with the accurate tracking of disease progression. Exclusion criteria for the healthy controls were: a history of neurological disorders that affect the brain or central nervous system, abnormal findings at general neurological examination and unwillingness to be informed of unexpected medical findings.

### Study design

For the present study, we included all ProPark participants (both PwPD and healthy controls), enrolled between September 2021 and September 2024, provided that at least two nights and days of accelerometer data was available. This left 464 PwPD and 105 healthy control participants to be included in the analysis comparing RAR between PwPD and healthy controls. A subgroup of PwPD was used to examine the associations between RAR and clinical factors. This subgroup consisted of PwPD with no missing data for the explanatory variables, leaving a total of 255 PwPD for this analysis (see Supplemental Material 1 for an overview of the number of missing values per variable).

We used data of the ProPark baseline visit that included a clinical assessment (i.e., physical and cognitive assessment) at the hospital. Subsequently, participants wore a wearable sensor containing an accelerometer and gyroscope (Axivity AX6; Axivity Ltd Newcastle, UK) measuring at 100 Hz with a range of +/- 8 g and +/- 2000 dps. The sensor was worn for seven days on the most affected wrist for PwPD with unilateral motor symptoms, and the non-dominant wrist for PwPD with bilateral motor symptoms and the healthy control group. During this week all participants filled out online questionnaires about the presence and severity of clinical symptoms, as detailed below.

### Demographics and clinical assessment

Age and gender were noted for each participant. Disease duration was based on the date of diagnosis as provided by the treating neurologist. Motor symptom severity and disease stage were assessed by a trained research associate using the Movement Disorders Society Unified Parkinson Disease Rating Scale part III (UPDRS-III),^17^ and Hoehn and Yahr (H&Y) scale,^18,19^ respectively. To assess cognitive impairment, the 11-item MoCA was used.^20^ Orthostatic hypotension (OH) was defined as a decrease in systolic blood pressure of ≥20 mmHg or a decrease in diastolic blood pressure of ≥10 mmHg, within three minutes of standing.^21^ The presence of OH was added as a binary variable. In addition to calculating OH, the percentage of PwPD with asymptomatic OH was determined by the absence of complaints (e.g., nausea, light headiness or problems with balance), after one and/or three minutes of standing.

During an interview, all drugs used by the participant were recorded. For each PwPD, the Levodopa equivalent daily dose (LEDD) was calculated (adapted from previous studies,^22,23^ as described in Supplemental Material 2). The use of sleep medication (i.e., clonazepam, oxazepam, diazepam, zolpidem, temazepam, and melatonin) was determined and included in subsequent analyses as a binary variable for all participants.

The subscales of the Scales for Outcomes in Parkinson's Disease Sleep (SCOPA-SLEEP) were used to evaluate nighttime sleep and daytime sleepiness.^24^ To assess REM sleep behavior disorder (RBD), the 10-item REM Sleep Behavior Disorder Screening Questionnaire (RBD-SQ) was used.^25^ Fatigue was evaluated by the Movement Disorders Society Unified Parkinson Disease Rating Scale part I (UPDRS-I), question 13.^17^ Urinary dysfunction was evaluated by the subscore of the Scales for Outcomes in Parkinson's Disease Autonomic questionnaire (SCOPA-AUT).^26^ Apathy, depression, and anxiety were evaluated using the 12-item Apathy Evaluation Scale for Parkinson's Disease (AES-12PD),^27^ the 21-item Beck Depression Inventory-II (BDI-II),^28^ and the 12-item Parkinson Anxiety Scale (PAS),^29^ respectively.

To address missing values within these questionnaires, the following rules were applied: in the case of RBD-SQ, instances where the questionnaire was completed by the patient, rather than their partner, any missing values were imputed as ‘no’ (K. Stiasny-Kolster, personal communication). For SCOPA-AUT, missing values were substituted by the mean score of the non-missing items when the number of missing items did not exceed six within the questionnaire.^26^ Similarly, missing values in the SCOPA-SLEEP questionnaire were imputed with the mean score of the non-missing items if there were less than two missing items.^24^

The study began including participants during the COVID-19 pandemic and during this time a decrease in physical activity due to social distancing and confinement was observed.^30^ Whether or not a home assessment took place during the pandemic was taken into consideration as a binary variable. The end date of the COVID-19 pandemic in this study is seen as February 25^th^ 2022, since the last of the contact-reducing regulations in the Netherlands were lifted on this date.^31^ If the COVID-19 end date was during the home assessment week, the value of this variable was considered missing. See Table 2 for a complete list of variables used.

### Rest-activity rhythm assessment

**Figure 1.**
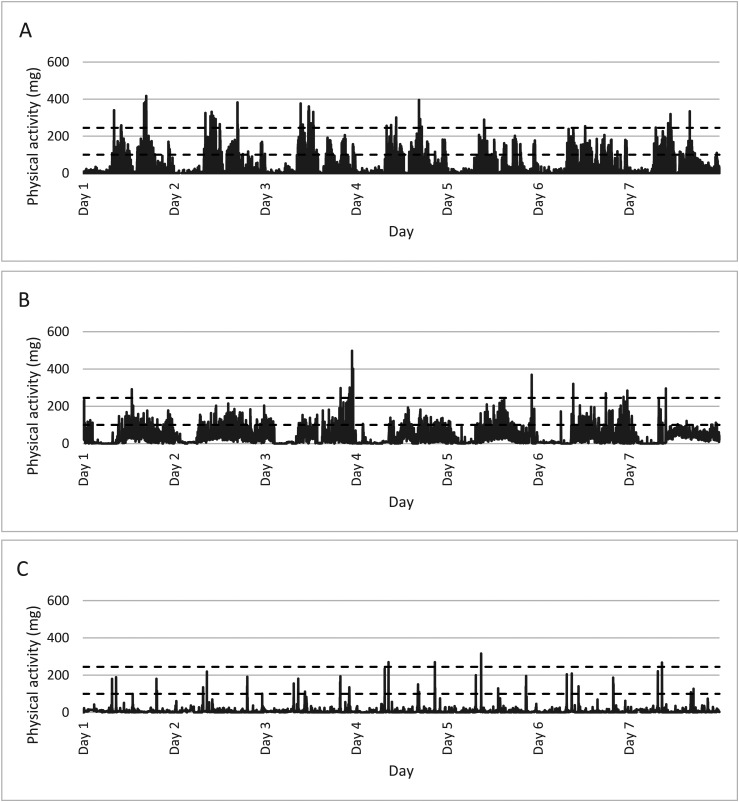
Physical activity levels (i.e., ENMO scores) over seven days for three participants. (A) represents a 64-year-old healthy control male, (B) represents a 64-year-old male with PD, H&Y: 2, UPDRS-III: 19, (C) represents a 67-year-old male with PD, H&Y: 2, UPDRS-III: 20. Black dotted lines indicate moderate (100 mg) and vigorous (245 mg) activity cut-offs. ENMO: Euclidean Norm Minus One; H&Y: Hoehn & Yahr stage; UPDRS-III: Unified Parkinson's disease Rating Scale part III total score.

To determine the RAR measures, the raw acceleration files from the wrist sensors were processed in R,^32^ using the open-source GGIR package version 3.1–4.^33^ Physical activity was quantified from the raw triaxial signals as the Euclidean Norm Minus One (ENMO), expressed in mg, and calculated in one minute epochs. This measure adjusts for gravitational effects by subtracting 1 g, and rounds any negative values up to zero.^34^ Figure 1 presents three examples illustrating the variability of ENMO scores across both PD patients and healthy control participants, with the black dotted lines indicating moderate and vigorous activity cut-offs.^35^ Despite the similarities in disease severity, the weekly patterns of physical activity in the two PD patients are notably different.

Based on ENMO values, three RAR related variables were calculated over the entire measurement period using the GGIR package.^33^ The first variable is relative amplitude, defined as a ratio calculated by dividing the difference in the sum of the activity during the most active ten hours and the least active five hours, by the sum of the activity level in these periods.^12,36^ This variable ranges from 0 to 1, where a higher value indicates having more distinct periods of rest and activity.^10,37^ Second, intradaily variability estimates the fragmentation of RAR, i.e., the changes in physical activity on an hourly basis throughout the days of measurement.^38^ This variable has a range from 0 to 2, with a higher value representing a more fragmented rhythm.^39^ Third, interdaily stability indicates the consistency of RAR between days.^38^ This variable has a range of 0 to 1, with a higher value representing a more constant rhythm over multiple days.^40^ The precise mathematical explanation of intradaily variability and interdaily stability can be found elsewhere.^38^

### Statistical analyses

To compare difference in RAR between PwPD and healthy controls, linear regression models were performed with RAR as dependent variable, corrected for age and gender. To analyze which clinical symptoms are associated with RAR in PwPD, we performed backward stepwise linear regression analysis with a stopping rule of *p* < 0.05. To analyze which clinical symptoms are associated with RAR in PwPD, we performed backward stepwise linear regression analysis with a stopping rule of p < 0.05, including the sixteen demographic and clinical predictors discussed earlier. A power analysis was conducted using G*Power 3.1.9.7 for a linear multiple regression (fixed model, R^2^ deviation from zero.^41,42^ As no similar prior studies were found, an effect size representing a small-to-medium effect size was chosen. The power analysis indicated a required sample size between 211 and 272, with f^2^ ranging from 0.07 to 0.09, sixteen predictors, α = 0.05, and Power = 0.80. Possible multicollinearity between the predictor variables was addressed by excluding variables exhibiting correlations exceeding 0.5, indicative of at least moderate correlation with other variables.^43^ The variable showing collinearity with the most variables is removed from the analysis. This allows the remaining variables to be included in the final model while avoiding multicollinearity. If two variables are equal, the variable with the lowest explained variance in a univariate regression model is removed from the analysis. To obtain standardized estimates, all variables were transformed to *z* scores prior to fitting the regression models. Bootstrapping was used to resample the data with replacement, providing a better view of the population's distribution.^44^ With the resampled data, the 95% confidence intervals (CI) are calculated. Results were considered statistically significant when zero was not included in this CI.^45^ This supports the robustness of the prediction models as the results remain significant, even in the resampled data.

## Results

### Rest-activity rhythm in people with Parkinson's disease compared to healthy controls

**Figure 2.**
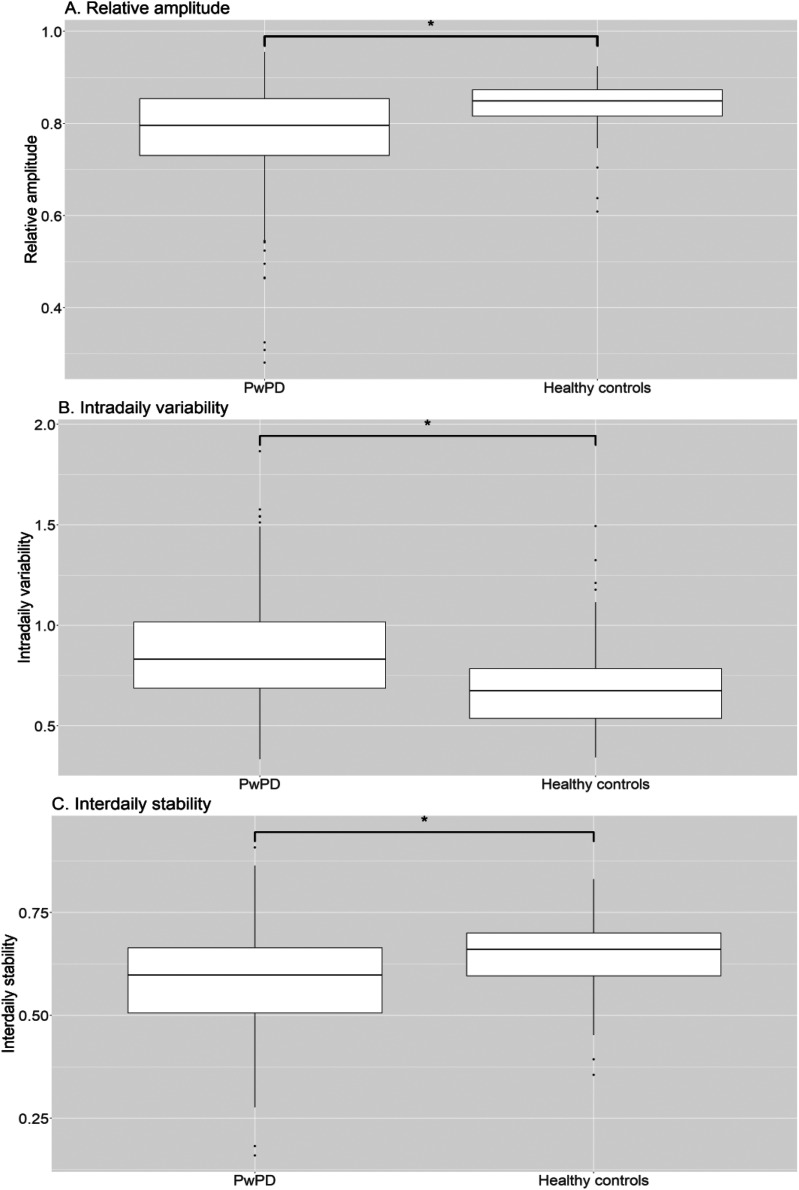
Group distributions of RAR in PwPD and healthy controls. Relative amplitude (A), intradaily variability (B), and interdaily stability (C). A significant group level difference between PwPD and Healthy Controls, corrected for age and gender, is indicated with an asterisk (*p < 0.001). PwPD: people with Parkinson's disease.

Age was similar in PwPD and healthy controls, and there were more males in the PwPD group (Table 1). All three RAR variables were significantly different in PwPD compared to healthy controls (Figure 2). In comparison to healthy controls, PwPD had lower relative amplitude (mean (SD) in PwPD: 0.783 (0.097); HC: 0.840 (0.050); *p* < 0.001), higher intradaily variability (mean (SD) in PwPD: 0.832 (0.252); HC: 0.697 (0.211); *p* < 0.001), and lower interdaily stability (mean (SD) in PwPD: 0.598 (0.117); HC: 0.664 (0.087); *p* < 0.001).

**Table 1.** Demographics of PwPD and healthy controls, and clinical characteristics of PwPD.

	PwPD (N = 464)	Healthy controls (N = 105)
*Demographic characteristics*		
Age in years, mean (SD)	67 (8)	67 (10)
Male, n (%)	304 (65.5)	45 (42.9)
*Clinical characteristics*		
Disease duration in years*	2.8 (1.1–5.5)	
UPDRS-III*	20 (13–30)	
H&Y stage, n (%)		
1	66 (14.2)	
2	376 (81.0)	
3	15 (3.2)	
4	2 (0.4)	
missing	5 (1.1)	
LEDD*	500 (300–750)	
*Accelerometer measurement*		
Number of days measured, n (%)		
2	3 (0.6)	0 (0.0)
3	6 (1.3)	1 (1.0)
4	3 (0.6)	0 (0.0)
5	13 (2.8)	4 (3.8)
6	72 (15.5)	16 (15.2)
7	367 (79.1)	84 (80.0)

All values are median (IQR), unless otherwise noted.

*Missing values for disease duration (n = 54), UPDRS-III (n = 7), and LEDD (n = 16) were excluded from calculating the median and interquartile range values.

PwPD: People with Parkinson's disease; PD: Parkinson's disease; UPDRS-III: Unified Parkinson's disease Rating Scale part III total score; H&Y: Hoehn & Yahr stage; LEDD: Levodopa Equivalent Daily Dose.

### Clinical symptoms associated with disturbances in rest-activity patterns in people with Parkinson's disease

The characteristics of the PwPD with no missing data in the explanatory variables (Supplemental Material 1) and a summary of the fifteen explanatory variables used in the analysis to examine the extent to which motor and non-motor symptoms are associated with RAR disruption are shown in Table 2. Depression was removed as predictor variable due to being moderately correlated with anxiety, apathy, fatigue, and nighttime sleep (Supplemental Material 3). With fifteen remaining predictors and a final sample consisting of N = 255, this analysis matches the minimum required sample size as determined by the power analysis.

**Table 2.** Characteristics of the PwPD, and summary of exploratory variables used in the analysis.

	PwPD (N = 255)
*Demographic characteristics*	
Age in years, mean (SD)	66 (8)
Male, n (%)	165 (64.7)
*Clinical characteristics*	
Disease duration in years*	2.7 (1.0–5.3)
UPDRS-III	19 (12–29)
H&Y stage, n (%)	
1	38 (14.9)
2	209 (82.0)
3	5 (2.0)
4	2 (0.8)
NA	1 (0.4)
LEDD	450 (300–720)
*Actigraphy measurement*	
Number of days measured, n (%)	
2	2 (0.8)
3	3 (1.2)
4	3 (1.2)
5	7 (2.7)
6	35 (13.7)
7	205 (80.4)
*Explanatory variables included in the stepwise regression models*	
Age in years, mean (SD)	66 (8)
Male, n (%)	165 (64.7)
Motor function (UPDRS-III)	19 (12–29)
LEDD	450 (300–720)
Cognitive impairment (MoCA)	27 (25–29)
Anxiety (PAS)	6 (3–10)
Apathy (AES-12PD)	25 (20–29)
Daytime sleepiness (SCOPA-SLEEP subscore)	3 (1–5)
Nighttime sleep (SCOPA-SLEEP subscore)	4 (2–6)
REM sleep behavior disorder (RBD-SQ)	2 (1–5.5)
Fatigue (UPDRS part 1 question 13)	1 (0.5–2)
Sleep medication, yes (n (%))	19 (7.5)
Orthostatic hypotension, yes (n (%))**	59 (23.1)
Urinary dysfunction (SCOPA-AUT subscore)	6 (4–8.5)
COVID-19 restrictions, yes (n (%))	20 (7.8)

Demographic and clinical characteristics of the PwPD included in the regression analysis, and the exploratory variables used in this. All values are median (IQR), unless otherwise noted.

*Missing values for disease duration (n = 35) were excluded from calculating the median and interquartile range values.

** Of the 59 PwPD with orthostatic hypotension, 50 (84.7%) is asymptomatic.

PwPD: people with Parkinson's disease; UPDRS-III: Unified Parkinson's Disease Rating Scale part III total score; H&Y: Hoehn & Yahr; LEDD: Levodopa equivalent daily dose; MoCA: Montreal Cognitive Assessment; PAS: Parkinson Anxiety Scale; AES-12PD: Apathy Evaluation Scale for Parkinson's Disease; SCOPA-SLEEP: Scales for Outcomes in Parkinson's Disease – Sleep; RBD-SQ: REM Sleep Behavior Disorder Screening Questionnaire; SCOPA-AUT: Scales for Outcomes in Parkinson's Disease – Autonomic.

**Figure 3.**
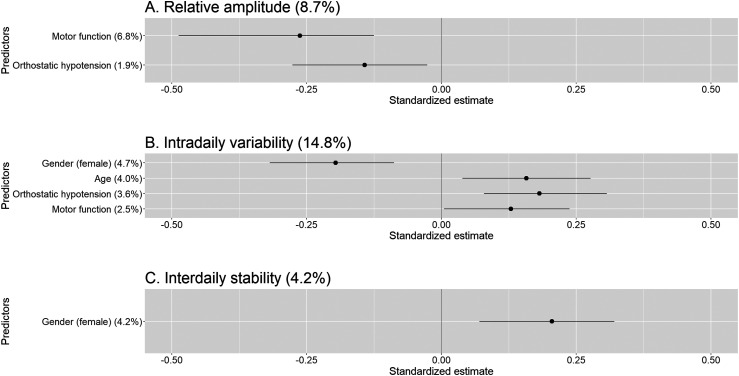
Predictors significantly associated with rest-activity rhythm. Standardized estimates (dots) with 95% confidence intervals (horizontal lines) of the final stepwise linear regression models are shown for all three RAR variables, i.e., relative amplitude (A), intradaily variability (B), and interdaily stability (C) in PwPD. Percentage of total explained variance and predictor specific explained variance of the RAR variables is provided between round brackets. PwPD: people with Parkinson's disease.

For PwPD, more severe motor symptoms (β= −0.262, 95% CI = [-0.487,-0.125], R²= 6.8%) and the presence of OH (β= −0.142, 95% CI = [-0.276,-0.026], R²= 1.9%) were associated with lower relative amplitude. More severe motor symptoms (β= 0.129, 95% CI = [0.005,0.238], R²= 2.5%), the presence of OH (β= 0.182, 95% CI = [0.079,0.307], R²= 3.6%) and higher age (β= 0.158, 95% CI = [0.039,0.277], R²= 4.0%) were associated with higher intradaily variability, while female gender was associated with lower intradaily variability (β= −0.196, 95% CI = [-0.318,-0.088], R²= 4.7%). Female gender was associated with higher interdaily stability (β= 0.205, 95% CI = [0.071,0.321], R²= 4.2%). The final models are visualized in Figure 3.

## Discussion

Our study showed that, compared to healthy controls, PwPD have a RAR characterized by lower relative amplitude, higher intradaily variability, and lower interdaily stability. In addition to higher age and male gender, dysregulation of RAR in PwPD was significantly associated with more severe motor symptoms and the presence of OH.

Higher motor symptom severity was associated with lower relative amplitude, which is mainly driven by a lower physical activity during the day as opposed to higher activity during the night (Supplemental Material 4). The association between more severe motor impairment and diurnal physical activity is in line with earlier studies showing that motor impairment influences the performance of ADL and exercise capacity.^46^ Additionally, more severe motor impairment was associated with higher intradaily variability, i.e., more fragmentation in physical activity throughout the day. We hypothesized that this fragmentation could be related to motor fluctuations, as it is a likely cause for limiting physical activity.^47^ However, a post-hoc analysis (Supplemental Material 5) revealed no significant association between intradaily variability and UPDRS-IV scores. Yet, the generalizability of the post-hoc results is questionable because the number of PwPD with significant motor fluctuations included in the analysis was limited. An alternative explanation for the relation between motor symptom severity and intradaily variability could be that fatigue, which is associated with motor function,^48^ leads to the need for more breaks throughout the day. However, fatigue was not one of the final predictors of RAR. In this study, fatigue was assessed by question 13 of UPDRS-I. Notably, individual items of a rating scale such as the UPDRS-I are adequate to globally identify the presence of a certain symptom but insufficient to formally quantify it.^49^ This may explain the lack of association between fatigue and RAR in this study. While motor symptom severity (i.e., UPDRS III sum score) was a significant predictor for both relative amplitude and intradaily variability, it is unclear what the individual associations of specific motor symptoms are with RAR parameters. An exploratory analysis was conducted to explore the associations between both relative amplitude and intradaily variability, and the three motor symptoms domains of the UPDRS-III (i.e., bradykinesia/rigidity, tremor, postural instability/gait difficulty) (Supplemental Material 6). The analysis revealed that the bradykinesia/rigidity and postural instability/gait difficulty domains are most strongly linked with RAR. Worse scores in these domains are associated with lower relative amplitude and higher intradaily variability. This finding is consistent with earlier studies showing that PwPD with postural instability/gait difficulties have lower scores in activities of daily living than tremor-dominant PwPD.^50^

To our knowledge, this is the first study to find an association between OH and RAR in PwPD. OH in PD is thought to be the result of disturbances in pathological processes, such as norepinephrine transmitter system,^51,52^ and side effects of dopaminergic medication.^21^ Deficiencies in norepinephrine could be a possible biological mechanism behind the association of OH and RAR as it is also linked to processes closely related to the circadian rhythm and RAR such as arousal and the sleep-wake cycle.^51^ With this cross-sectional study, we cannot definitively determine the direction of the relationship between OH and RAR. However, as only extreme levels of physical inactivity can only lead to OH, i.e., after multiple days of being bed bound,^53^ a more likely direction of the relationship is that OH influences physical activity and disturbances in RAR. This is also indicated by studies that found that the presence of OH was associated with impairments in the performance of ADL, even if OH is asymptomatic.^54^ In the present study, OH was associated with RAR, even with 84.7% of the PwPD with OH being asymptomatic. This highlights the need for regular evaluation of OH, even when symptoms are not present.

Women had a lower intradaily variability and higher interdaily stability than men. The relationship between gender and RAR has been extensively studied in older healthy adults. Consistent with our results, these studies have shown that women often have lower intradaily variability,^55^ and higher interdaily stability.^39^ Sociocultural factors have been proposed as a possible reason for the gender differences in RAR.^39^ For instance, women may be more often involved in regular household activities and thus hold a more regular physical activity schedule.^39^ We also found that older age was associated with higher intradaily variability. The relationship between age and intradaily variability remains unclear in healthy populations. One study reports similar results, suggesting that clinical conditions that are more common with age, may disrupt circadian rhythms such as RAR.^56^ In contrast, another study found that older adults had lower intradaily variability. However, this study did not report information on morbidity, making it difficult to determine whether this may have been the cause of the difference in results between these studies. While age- and gender-related behavioral characteristics are plausible explanations for these findings, biological differences may also contribute to these associations. RAR is a behavioral expression of circadian rhythms, and sex- and age-related differences, such as anatomical or functional brain differences, are known to play a role in these rhythms.^57,58^

The observed disruption of RAR in PwPD compared to healthy controls is in line with previous work.^10,11,13^ Only one study was found that used the same RAR variables as this study. Yet, that study did not show differences in relative amplitude and interdaily stability when comparing PwPD to healthy controls, and only a difference in intradaily variability.^10^ Even though no significant results were found for relative amplitude and interdaily stability in the previous study, the direction of the effect is similar. The difference between PwPD and healthy controls indicates that there are disease-specific aspects that play a role in RAR disturbances. Yet, interdaily stability does not have any associations with PD-related clinical symptoms. Possibly, the difference between PwPD and healthy controls is not only PD specific but could also be due to differences in social factors. For example, work plays a big part in the consistency of RAR during the week, yet PwPD are more often unemployed or retire earlier than healthy adults.^59^ Lacking the structure of a workday may possibly be an explanation for the difference that is found in interdaily stability between PwPD and healthy controls.

The explained variance of clinical variables that were associated with changes in RAR was modest, ranging from 4.2% to 14.8%. To our knowledge, we are the first to examine associations between RAR and clinical variables in PwPD, making it difficult to contextualize our findings as either high or low. Notably, RAR changes are only partially explained by PD-related and demographic factors. Other unknown biological, behavioral, and social factors need to be further explored.^55^ As mentioned earlier, work can have an influence on the regularity of RAR and the overall physical activity levels over a day.^60^ Furthermore, reduced social support and social interaction also play a role in the physical activity of older adults.^39,61^ Many social and environmental factors have already been shown to affect RAR in older adults, and are therefore likely contributors to the unexplained variance in RAR among PwPD. For example, work status plays a key role. Maintaining a work schedule can promote a regular daily rhythm through consistent timing of activities, leading to greater interdaily stability.^62^ Conversely, retirement may reduce overall physical activity if this is not compensated by increased leisure activity.^60^ Beyond employment, social participation through volunteering, sports, hobby clubs, or neighborhood associations is also linked to higher levels of physical activity.^63^ Reduced social interaction negatively impacts the physical activity of older adults.^39,61^ Additionally, environmental factors such as the current season influence activity levels. Increased physical activity is typically observed during the summer months.^58^ Together, these social and contextual factors warrant further investigation to better understand their role in the unexplained variance of RAR parameters in PwPD.

Given the wide variety of factors associated with RAR, it was not expected that high explained variance would be found with demographic and PD-related factors alone. However, despite the known impact of COVID-19 restrictions on physical activity levels in the overall population,^30^ the presence of a COVID-19 lockdown was not a significant predictor in any of the models, demonstrating that PD-related factors have a greater impact on the daily activity patterns of PwPD than COVID-19.

### Clinical relevance

RAR is an example of a circadian rhythm because it measures activity over a 24-h period.^5^ In PD, endogenous oscillators that help regulate circadian rhythms are disturbed.^64^ As a result, exogenous oscillators, such as physical activity, become particularly important for maintaining a robust rhythm.^65^ Specifically PwPD displaying an disturbed RAR may thus benefit from advice and interventions to improve regular physical activity. For example, establishing a regular wake-up time and regular exercise have a positive effect on regulating the circadian regulation.^65^ Although many PD-related symptoms from the motor, sleep, autonomic, and neuropsychiatric domains were expected to play a role in RAR disruption, only motor symptom severity and the presence of OH remained as significant PD-related factors that are associated with disturbances in RAR in this study. Treating OH is important to reduce symptom burden and manage quality of life.^21^ Hypothetically, optimizing the clinical treatment of motor symptoms and OH, even when symptoms are not present, may reduce RAR disturbances in PwPD. Also, monitoring RAR in PwPD may help identify those who are likely to have OH, including asymptomatic patients, who should therefore be evaluated by a clinical. However, more research is needed to determine if RAR can serve as a predictive digital biomarker for OH, whether alleviating motor symptoms and OH can actually improve RAR, and whether improving RAR leads to improved quality of life.

### Limitations

The analyses in this study were performed on cross-sectional data, so it is not possible to draw conclusions about the causality of the associations found between PD-related variables and RAR. Further research, including longitudinal and intervention studies, is needed to learn more about the causality of these associations. In addition, the generalizability of the results is limited because most of the PwPD included in this study had similar H&Y, with 81.1% of the participants in H&Y stage 2. To improve generalizability, further studies are needed in other cohorts with higher disease severity. As mentioned above, this study exclusively focused on demographic and PD-related factors. Other potentially relevant factors related to RAR were beyond the scope of this study. Although these factors, such as social and work environment, have been studied in healthy individuals,^55,66^ their relationship to RAR may be disturbed in PwPD. Future research should address these behavioral factors in relation to PD and RAR, for example through qualitative studies that explore the behavioral and contextual influences on daily living patterns in PwPD.

## Conclusion

PwPD are less active during the day, have more fragmentation of activity within days, and more variance in activity over multiple days compared to healthy individuals. Dysregulation of RAR in PwPD is associated with the presence of OH, more severe motor symptoms, male gender and older age. These findings highlight the importance of addressing motor symptoms and both symptomatic and asymptomatic OH in clinical practice, as it may be beneficial to improve RAR and ultimately mobility in PwPD.

## Supplemental Material

sj-docx-1-pkn-10.1177_1877718X251388890 - Supplemental material for Disturbed rest-activity rhythm in Parkinson's disease: Associations with motor severity and orthostatic hypotension Supplemental material, sj-docx-1-pkn-10.1177_1877718X251388890 for Disturbed rest-activity rhythm in Parkinson's disease: Associations with motor severity and orthostatic hypotension by Manon D Mijnsbergen, Vasileios Exadaktylos, Jacobus J van Hilten, Dagmar H Hepp and Roel HA Weijer in Journal of Parkinson's Disease
