# Supplementary material for: Disturbed rest-activity rhythm in Parkinson's disease: Associations with motor severity and orthostatic hypotension
Source: J Parkinsons Dis. 2025 Oct 21:1877718X251388890. Online ahead of print. doi: 10.1177/1877718X251388890 (PMC13347550; doi:10.1177/1877718X251388890)
Supplement: Supplementary file 1 [file sj-docx-1-pkn-10.1177_1877718X251388890.docx]

**Supplemental Material**

**Disturbed rest-activity rhythm in Parkinson’s disease: Associations with motor severity and orthostatic hypotension**

**Supplemental Material 1. Missing values of predictor variables**

| **Variable** | **Number of participants with missing values, n (%)**  **(total number of participants: N = 464)** |
| --- | --- |
| Age | 0 (0%) |
| Gender | 0 (0%) |
| Motor function (UPDRS-III) | 7 (1.5%) |
| LEDD | 0 (0%) |
| Cognitive impairment (MoCA) | 8 (1.7%) |
| Anxiety (PAS) | 100 (21.6%) |
| Apathy (AES-12PD) | 99 (21.3%) |
| Depression (BDI-II) | 139 (30.0%) |
| Daytime sleepiness (SCOPA-SLEEP subscore) | 107 (23.1%) |
| Nighttime sleep (SCOPA-SLEEP subscore) | 90 (19.4%) |
| REM sleep behavior disorder (RBD-SQ) | 75 (16.2%) |
| Fatigue (UPDRS-I, question 13) | 103 (22.2%) |
| Sleep medication | 0 (0%) |
| Orthostatic hypotension | 0 (0%) |
| Urinary dysfunction (SCOPA-AUT subscore) | 103 (22.2%) |
| COVID-19 restrictions* | 3 (1%) |

* Information COVID-19 restrictions was considered missing when restrictions were removed during the week in which the measurement was taking place.

UPDRS-III: Unified Parkinson’s Disease Rating Scale part III; LEDD: Levodopa equivalent daily dose; MoCA: Montreal Cognitive Assessment; PAS: Parkinson Anxiety Scale; AES-12PD: Apathy Evaluation Scale for Parkinson’s Disease; BDI-II: Beck Depression Inventory-II; SCOPA-SLEEP: Scales for Outcomes in Parkinson’s Disease – Sleep; RBD-SQ: REM Sleep Behavior Disorder Screening Questionnaire; UPDRS-I: Unified Parkinson’s Disease Ration Scale part I; SCOPA-AUT: Scales for Outcomes in Parkinson’s Disease – Autonomic.

**Supplemental Material 2. Formula for calculation of Levodopa Equivalent Daily Dose (LEDD)**

To calculate LEDD, the medication doses were be multiplied with the conversion factor given in the table below and added together. In addition, the following rules are applied.

- If a participant uses Levodopa + Carbidopa + Entacapon, Entacapon, or Tocapon: medication dose of all Levodopa medications are additionally multiplied by factor 0.2 and added to the final LEDD.
- If a participant uses Selegiline, Rasagiline, or Safinamide: medication dose of all Levodopa medications are additionally multiplied by factor 0.1 and added to the final LEDD.

| Medication Type | Conversion factor |
| --- | --- |
| Levodopa + Carbidopa (Sinemet) | 1 |
| Levodopa + Carbidopa retard (Sinemet CR) | 0.75 |
| Levodopa + Benserazide (Madopar) | 1 |
| Levodopa + Benserazide retard (Madopar CR) | 0.75 |
| Levodopa + Carbidopa + Entacapon (Stalevo / Corbilta) | 1 |
| Ropinirol (Requip / Adartrel) | 20 |
| Pramipexol ZOUT (Sifrol / Mirapexin / Glepark / Oprymea) | 100 |
| Pergolide (Permax) | 100 |
| Bromocriptine (Parlodel) | 10 |
| Apomorfine BOLUS (APO-go / Dacepton) | 10 |
| Rotigotine (Neupro pleister) | 30 |
| Amantadine (Symmetrel) | 1 |
|  |  |

**Supplemental Material 3. Correlation matrix of prediction variables**

|  | Age | Gender | LEDD | Sleep medication | COVID-19 restrictions | Cognitive impairment  (MoCA) | Motor functioning  (UPDRS-III) | Fatigue  (UPDRS-I, q13) | Apathy  (AES-12PD) |  |
| --- | --- | --- | --- | --- | --- | --- | --- | --- | --- | --- |
| Age | 1 | -0.152 | -0.101 | -0.071 | 0.073 | -0.255 | 0.173 | 0.012 | -0.255 |  |
| Gender | -0.152 | 1 | -0.022 | -0.019 | 0.089 | 0.146 | -0.188 | 0.207 | 0.114 |  |
| LEDD | -0.101 | -0.022 | 1 | 0.194 | 0.061 | 0.053 | 0.113 | 0.142 | -0.122 |  |
| Sleep medication | -0.071 | -0.019 | 0.194 | 1 | -0.082 | -0.036 | -0.021 | 0.148 | -0.117 |  |
| COVID-19 restrictions | 0.073 | 0.089 | 0.061 | -0.082 | 1 | -0.076 | 0.175 | 0.096 | 0.031 |  |
| Cognitive impairment (MoCA) | -0.255 | 0.146 | 0.053 | -0.036 | -0.076 | 1 | -0.253 | -0.042 | 0.141 |  |
| Motor functioning (UPDRS-III) | 0.173 | -0.188 | 0.113 | -0.021 | 0.175 | -0.253 | 1 | 0.048 | -0.041 |  |
| Fatigue (UPDRS-I, q13) | 0.012 | 0.207 | 0.142 | 0.148 | 0.096 | -0.042 | 0.048 | 1 | -0.257 |  |
| Apathy (AES-12PD) | -0.255 | 0.144 | -0.122 | -0.117 | 0.031 | 0.141 | -0.041 | -0.257 | 1 |  |
| Anxiety (PAS) | 0.001 | 0.284 | 0.153 | 0.213 | 0.042 | -0.042 | -0.045 | 0.428 | -0.398 |  |
| Depression (BDI-II) | 0.010 | 0.151 | 0.186 | 0.186 | 0.026 | -0.030 | -0.002 | **0.523** | **-0.590** |  |
| Nighttime sleep (SCOPA-SLEEP subscore) | -0.136 | 0.282 | 0.309 | 0.159 | 0.029 | 0.159 | -0.033 | 0.372 | -0.215 |  |
| Daytime sleepiness (SCOPA-SLEEP subscore) | 0.198 | 0.026 | 0.119 | -0.004 | 0.038 | -0.062 | 0.112 | 0.227 | -0.206 |  |
| REM sleep behavior disorder (RBD-SQ) | 0.134 | -0.025 | 0.210 | 0.157 | 0.066 | -0.063 | -0.024 | 0.126 | -0.257 |  |
| Urinary dysfunction (SCOPA-AUT subscore) | 0.303 | 0.015 | 0.092 | 0.023 | 0.059 | -0.095 | 0.107 | 0.242 | -0.371 |  |
| Orthostatic hypotension | 0.207 | 0.033 | 0.016 | -0.003 | 0.115 | -0.028 | -0.017 | 0.043 | -0.178 |  |
|  |  |  |  |  |  |  |  |  |  |  |

**Supplemental Material 4. Supplementary analysis showing comparison between Parkinson and control group of the mean activity in the most active ten hours and least active 5 hours**

|  | Anxiety  (PAS) | Depression  (BDI-II) | Nighttime sleep  (SCOPA-SLEEP subscore) | Daytime sleep  (SCOPA-SLEEP subscore) | REM sleep behavior disorder  (RBD-SQ) | Urinary dysfunction  (SCOPA-AUT subscore) | Orthostatic hypotension |
| --- | --- | --- | --- | --- | --- | --- | --- |
| Age | 0.001 | 0.010 | -0.136 | 0.198 | 0.134 | 0.303 | 0.207 |
| Gender | 0.284 | 0.151 | 0.282 | 0.026 | -0.025 | 0.015 | 0.033 |
| LEDD | 0.153 | 0.186 | 0.309 | 0.119 | 0.210 | 0.092 | 0.016 |
| Sleep medication | 0.213 | 0.186 | 0.159 | -0.004 | 0.157 | 0.023 | -0.003 |
| COVID-19 restrictions | 0.042 | 0.026 | 0.029 | 0.038 | 0.066 | 0.059 | 0.115 |
| Cognitive impairment (MoCA) | -0.042 | -0.030 | 0.159 | -0.062 | -0.063 | -0.095 | -0.028 |
| Motor functioning (UPDRS-III) | -0.045 | -0.002 | -0.033 | 0.112 | -0.024 | 0.107 | -0.017 |
| Fatigue (UPDRS-I, q13) | 0.428 | **0.523** | 0.372 | 0.227 | 0.126 | 0.242 | 0.043 |
| Apathy (AES-12PD) | -0.398 | **-0.590** | -0.215 | -0.206 | -0.257 | -0.371 | -0.178 |
| Anxiety (PAS) | 1 | **0.696** | 0.343 | 0.113 | 0.273 | 0.289 | 0.017 |
| Depression (BDI-II) | **0.696** | 1 | **0.513** | 0.202 | 0.285 | 0.367 | 0.082 |
| Nighttime sleep (SCOPA-SLEEP subscore) | 0.343 | **0.513** | 1 | 0.226 | 0.219 | 0.222 | 0.077 |
| Daytime sleepiness (SCOPA-SLEEP subscore) | 0.113 | 0.202 | 0.226 | 1 | 0.162 | 0.204 | -0.071 |
| REM sleep behavior disorder (RBD-SQ) | 0.273 | 0.285 | 0.219 | 0.162 | 1 | 0.261 | 0.126 |
| Urinary dysfunction (SCOPA-AUT subscore) | 0.289 | 0.367 | 0.222 | 0.204 | 0.261 | 1 | 0.210 |
| Orthostatic hypotension | 0.017 | 0.082 | 0.077 | -0.071 | 0.126 | 0.210 | 1 |

A supplementary analysis is done to see if the significant difference in relative amplitude between the PwPD and healthy controls is because of a significant difference of the most active ten hours during the day, or the least active five hours during the night. Supplemental Figure 1 shows a visualization of the comparison between PwPD and healthy controls for both variables. For the mean physical activity during the most active ten hours, there is a significant difference between PwPD and controls (mean (SD) in PwPD: 30.872 mg (14.273 mg); HC: 39.829 mg (11.556 mg); *p* < 0.001). No significant difference is found for the mean activity during the least active five hours between PwPD and healthy controls (mean (SD) in PwPD: 3.330 mg (1.298 mg); HC: 3.278 mg (0.817 mg); *p* < 0.842). Indicating that the significant difference in relative amplitude between PwPD and healthy controls can be primarily attributed to alterations in diurnal activity levels.

**Supplemental Figure 1. Group distributions of RAR in PwPD and Healthy Controls.** Physical activity during the most active ten hours (A), and the physical activity during the least active five hours (B). A significant group level difference between PwPD and Healthy Controls, corrected for age and gender, is indicated with an asterisk (**p* < 0.05). ENMO: Euclidean Norm Minus One; PwPD: people with Parkinson’s disease.

**Supplemental Material 5. Supplementary analysis comparing intradaily variability, and motor fluctuation determined via UPDRS-IV.**

A possible explanation given for the association between motor function and intradaily variability is the presence of motor fluctuation. To possibly support this explanation, this supplementary analysis compares intradaily variability with UPDRS-IV question 3, i.e., time spent in off phase, and question 4, i.e., functional influence of motor fluctuations. The results are visualized in Supplemental Figure 2. For both questions, a one-way ANOVA is done to check for significant difference in intradaily variability between the scores for the UPDRS-IV questions. No significant difference was found for question 3 (F = 0.001, p = 0.98) and question 4 (F = 0.397, *p* = 0.529).

**Supplemental Figure 2. Comparison of intradaily variability and UPDRS-IV.** Question 3, i.e., time spent in off phase (A) and question 4, i.e., functional influence of motor fluctuations (B). 9 participants were excluded from analysis due to missing values in question 3 or 4 in the UPDRS-IV questionnaire.

UPDRS-IV: Unified Parkinson Rating Scale part 4.

**Supplemental Material 6. Supplementary analysis into the individual contributions of motor symptoms to RAR parameters**

Since the MDS-UPDRS-III score was a significant predictor of both relative amplitude and intradaily variability, an exploratory analysis was conducted to examine the individual contributions of specific motor symptom domains. Three subscores were calculated from the MDS-UPDRS: bradykinesia/rigidity (items 3.3, 3.4, 3.5, 3.6, 3.7, and 3.8), tremor (items 2.10, 3.15, 3.16, 3.17, and 3.18) and postural instability/gait difficulty (items 2.12, 2.13, 3.10, 3.11, and 3.12).^1, 2^ Separate linear regression models were performed for relative amplitude and intradaily variability, each including the three subscores as predictors.

Higher bradykinesia/rigidity (β=−0.0024, SE=0.0009, p=0.009) scores and postural instability/gait difficulty scores (β=−0.0100, SE=0.0039, p=0.011) were significantly associated with a lower relative amplitude. No significant association was found for tremor (β=0.0015, SE=0.0039, p=0.3358).

Intradaily variability was significantly higher with higher bradykinesia/rigidity (β=0.0048, SE=0.0341, p=0.0359) and postural instability/gait difficulty (β=0.0221, SE=0.0100, p=0.0285) scores. in contrast, intradaily variability was significantly lower when the tremor score was higher (β=-0.0092, SE=0.0040, p=0.0285).

**References**

1.	Stebbins GT, Goetz CG, Burn DJ, et al. How to identify tremor dominant and postural instability/gait difficulty groups with the movement disorder society unified Parkinson's disease rating scale: Comparison with the unified Parkinson's disease rating scale. *Mov Disord* 2013; 28: 668-670.

2.	Poston KL, Ua Cruadhlaoich MAI, Santoso LF, et al. Substantia nigra volume dissociates bradykinesia and rigidity from tremor in Parkinson’s disease: a 7 Tesla imaging study. *J Parkinsons Dis* 2020; 10: 591-604.
